# Joint line position change in primary total knee arthroplasty: a radiographic analysis comparing conventional and robotic techniques

**DOI:** 10.1007/s00264-023-06031-1

**Published:** 2023-11-09

**Authors:** Nicholas F. Cozzarelli, Cristian A. DeSimone, Taylor D’Amore, Matthew B. Sherman, Jess H. Lonner

**Affiliations:** Department of Orthopedic Surgery, Rothman Orthopaedic Institute at Thomas Jefferson University, 125 South 9th Street Suite 1000, Philadelphia, PA 19107 USA

**Keywords:** Total knee arthroplasty, TKA, Joint line, Elevation, Distalization, Robotic assisted, Conventional

## Abstract

**Purpose:**

Joint line (JL) position change in total knee arthroplasty (TKA) may alter knee biomechanics and impact function. The purpose of this study was to compare the change in JL position between robotic-assisted TKA (RA-TKA) and conventional TKA (C-TKA).

**Methods:**

A retrospective, radiographic analysis was conducted of patients who underwent RA-TKA and C-TKA to compare JL position change. JL position was measured in consecutive RA-TKAs and C-TKAs performed by four fellowship-trained arthroplasty surgeons. Statistical analysis was done utilizing *t*-tests and Mann Whitney *U* tests, with statistical significance being defined as a *p* value < 0.05.

**Results:**

Six hundred total RA-TKAs and 400 total C-TKAs were included in the analysis. There were no significant differences in patient baseline characteristics such as body mass index, range of motion, and tibiofemoral coronal alignment. RA-TKAs were associated with an average of 0.04 (2.2) mm JL position change, and C-TKAs were associated with an average 0.5 (3.2) mm JL position change (*p* = 0.030). There were inter-surgeon differences when comparing the change in JL position for RA-TKAs and C-TKAs between the four participating surgeons.

**Conclusion:**

RA-TKA leads to better preservation of the JL position than C-TKA, and this seems to be dependent on the arthroplasty surgeon’s preferences and techniques during TKA. Whether this statistically significant difference is clinically relevant needs to be further investigated.

## Introduction

Total knee arthroplasty (TKA) is growing in use worldwide, and strategies to optimize functional outcomes and durability are important. Joint line (JL) position preservation is a goal for TKA because of how it can affect knee kinematics and outcomes [1, 2]. Elevation of the JL impacts knee biomechanics by increasing mid-flexion laxity and altering posterior cruciate ligament (PCL) tension [3, 4], which may negatively impact post-operative outcomes [5]. Several studies have shown significantly lower patient-reported outcome measures with excessive JL elevation [3–7]. Inferior outcomes have also been found to be associated with JL distalization in revision TKA, although its impact in primary TKA is less clear [8].

Positioning of the JL in TKA varies based on implant selection, surgical technique, PCL handling, inherent errors with conventional instruments, and severity of pre-operative flexion contractures [9, 10]. Receiving computerized information on distal femoral resection thickness, limb alignment, joint gaps, and implant orientation may facilitate re-establishment of the JL height while achieving a balanced knee in robotic-assisted TKA (RA-TKA) [11–14]. While component alignment has been studied extensively in RA-TKA, there have been fewer studies investigating JL restoration [11–16]. Several studies have found that RA-TKA leads to better preservation of the JL than conventional TKA (C-TKA), but these were performed on small sample sizes with less stringent inclusion criteria, such as the inclusion of patients with flexion contractures and tibiofemoral alignment deformities up to as much as 30°, which can impact JL position necessary to balance the extension and flexion gaps [12, 13, 15, 17].

The primary purpose of this study is to conduct a radiographic analysis of pre-operative and post-operative JL position in RA-TKA and C-TKA. Additionally, the secondary purpose is to compare whether there are differences in JL position change between the participating surgeons when using robotic-assisted or conventional techniques. We hypothesize that utilizing robotic-assistance will better maintain the pre-operative JL position and that there will be no significant differences in JL position changes between the surgeons.

## Materials and methods

### Study design

This study was a single-institution, retrospective radiographic analysis of patients who underwent primary TKA for osteoarthritis via a conventional or robot-assisted technique. TKAs were performed by four fellowship-trained, high volume arthroplasty surgeons. The C-TKAs were performed using a mechanical alignment technique, and the RA-TKAs were performed utilizing a restricted kinematic alignment algorithm. Despite different techniques, the goal was to achieve a balanced knee, by clinical assessment for C-TKAs, and by clinical assessment and intraoperative quantitative laxity evaluation for RA-TKAs. The JL position change from pre-operative to post-operative for 600 consecutive RA-TKAs and 400 consecutive C-TKAs were compared. This study was approved by an institutional review board (IRB).

### Inclusion and exclusion criteria

Patients were included if they underwent primary TKA via conventional methods or robotic-assistance and had pre-operative and post-operative weight-bearing anteroposterior (AP) radiographs. Patients were included only if they had less than a 10° flexion contracture and less than 10° of tibiofemoral coronal varus alignment pre-operatively. Patients undergoing revision or conversion arthroplasty, and those with isolated lateral compartment arthritis or recurvatum were excluded.

### Data collection

Patient characteristic information were obtained from our prospectively maintained institutional database. Pre-operative electronic medical records and standing pre-operative AP radiographs were manually reviewed to determine range of motion (ROM) and coronal tibiofemoral alignment. Implant type and design was obtained from retrospective review of operative records. The identical implant system was utilized for all TKAs. Picture Archiving and Communication Software (PACS) was utilized to access the radiographs.

The adductor tubercle (AT) method [18] for measuring JL position was utilized to analyze both pre-operative to post-operative AP radiographs. These measurements were compared to determine JL position changes after RA-TKA and C-TKA. The measurements were done in duplicate, with an interval of six weeks, by two independent reviewers (N.C. and C.D.) who were blinded to the surgical technique when measuring. Any measurement discrepancies were evaluated by a third reviewer (T.D.). The differences from pre-operative to post-operative JL position were determined. Positive values indicated JL distalization and negative values indicated elevation.

### Patient characteristics

There were no significant differences in sex, body mass index (BMI), or ROM between the RA-TKA and C-TKA groups (Table 1). Patients in the RA-TKA group were older than the C-TKA group (66.3 vs. 64.7; *p* = 0.006). The distribution of polyethylene inserts, femoral styles, and laterality are noted in Table 2.

**Table 1:** Patient baseline characteristics

	Total (*N* = 1000)	RA-TKA (*N* = 600)	C-TKA (*N* = 400)	*p* value
Age (years)	65.7 (9.13)	66.3 (9.07)	64.7 (9.15)	**0.006**
Sex:				0.417
Men	395 (39.5%)	230 (38.3%)	164 (41.0%)	
Women	605 (60.5%)	370 (61.7%)	236 (59.0%)	
Race:				0.285
White	648 (64.8%)	397 (66.2%)	251 (62.8%)	
Black	35 (3.5%)	18 (3.0%)	17 (4.3%)	
Hispanic	14 (1.4%)	5 (0.83%)	9 (2.3%)	
Other	21 (2.1%)	12 (2.0%)	9 (2.3%)	
Unreported	282 (28.2%)	168 (28.0%)	114 (28.5%)	
BMI (kg/m^2^)	30.6 (5.18)	30.5 (5.01)	30.8 (5.42)	0.418
Range of motion				
Extension	5.5 (3.7)	5.6 (3.8)	5.5 (3.6)	0.614
Flexion	113 (8.9)	114 (8.9)	113 (8.8)	0.233

Values given as mean (standard deviation) or number (%). Bold values indicate statistical significance

*RA-TKA* robotic-assisted total knee arthroplasty, *C-TKA* conventional total knee arthroplasty, *BMI* body mass index

**Table 2:** Operative information

	Total (*N* = 1000)	RA-TKA (*N* = 600)	C-TKA (*N* = 400)	*p* value
Laterality:				0.858
Right	523 (52.3%)	316 (52.7%)	207 (51.8%)	
Left	477 (47.7%)	284 (47.3%)	193 (48.2%)	
Polyethylene style:				** < 0.001**
CPS	84 (8.4%)	58 (9.7%)	26 (6.5%)	
MC with PCL Retained	130 (13%)	74 (12.3%)	56 (14%)	
MC with PCL Resected	110 (11%)	95 (15.8%)	15 (3.8%)	
CR	102 (10.2%)	61 (10.2%)	41 (10.3%)	
PS	376 (37.6%)	222 (37.0%)	154 (38.5%)	
UC	198 (19.8%)	90 (15.0%)	108 (27.0%)	
Femoral style:				0.620
CR	541 (54.1%)	320 (53.3%)	221 (55.3%)	
PS	459 (45.9%)	280 (46.7%)	179 (44.7%)	

Values given number (%). Bold values indicate statistical significance

*CPS* constrained posterior-stabilized, *MC* medial-congruent, *CR* posterior cruciate retaining, *PS* posterior stabilized, *UC* ultra-congruent, *CR* cruciate retaining

### Statistical analysis

A priori power analysis was performed, revealing a need for a total of 58 patients, with at least 29 patients in each group [17]. Descriptive analyses were performed for the full cohort. A multivariate analysis was performed to account for demographic variables and differences in implant types (cruciate-retaining (CR), ultra-congruent (UC), medial-congruent (MC), posterior-stabilized (PS), constrained-posterior-stabilized (CPS)). The PCL was resected in all cases in which a UC, PS, and CPS insert were used. With the MC insert, the PCL can be saved or resected depending on surgeon preference and intra-operative needs for soft tissue balancing. In this study, the PCL was retained in 54.2% and resected in 45.8% of cases using an MC insert. The PCL was saved in all cases in which a CR insert was used. Continuous data was compared using either *t*-test or Mann–Whitney *U* tests for the RA-TKA and C-TKA groups and ANOVA or Kruskal–Wallis when comparison were made between three or more groups for the sub-analyses. *p* Values less than 0.05 were deemed statistically significant. All statistical analyses were performed using R Studio (Version 3.6.3, Vienna, Austria).

## Results

### Radiographic measurements

There were no significant differences in pre-operative tibiofemoral coronal alignment between groups (Table 3). The RA-TKA group had an average change of − 0.04 (3.3) mm in JL position, whereas the C-TKA group had an average change of − 0.5 (3.2) mm in JL position (*p* = 0.030). In total, 56.2% of patients experienced JL elevation, 7.2% experienced no change, and 36.6% experienced JL distalization, with no significant differences in the directionality between groups. In the RA-TKA group, less than 15% experienced more than 4 mm of JL elevation compared to over 20% in the C-TKA group (*p* = 0.788); almost 26% and 30% of RA-TKAs and C-TKAs, respectively, had more than 4 mm of JL distalization (*p* = 0.417). Among the knees in which the JL was elevated, the RA-TKA group had an mean elevation of 2.2 (2.0) mm, which was significantly less than 2.7 (1.9) mm of for the C-TKA group (*p* < 0.001). Among the knees in which the JL was distalized, there was no significant difference in the amount of distalization between groups (3.2 mm vs 2.9 mm) (Table 3).

**Table 3:** Radiographic measurements

	Total (*N* = 1000)	RA-TKA (*N* = 600)	C-TKA (*N* = 400)	*p* value
Preoperative tibiofemoral coronal varus alignment	4.43 (3.5)	4.41 (3.4)	4.45 (3.5)	0.816
Preoperative JL position	51.2 (6.0)	51.4 (5.9)	50.9 (6.2)	0.292
Postoperative JL position	51.0 (6.2)	51.3 (6.2)	50.4 (6.2)	**0.036**
*p* value	0.028	0.735	0.002	
JL position change	− 0.23 (3.2)	− 0.04 (3.3)	− 0.50 (3.2)	**0.030**
Mean JL elevation	2.4 (2.0)	2.2 (2.0)	2.7 (1.9)	** < 0.001**
Mean JL distalization	3.1 (2.1)	3.2 (2.4)	2.9 (1.8)	0.448
JL change directionality:				0.602
Elevation	562 (56.2%)	332 (55.3%)	230 (57.5%)	
No change	72 (7.2%)	47 (7.8%)	25 (6.2%)	
Distalization	366 (36.6%)	221 (36.8%)	145 (36.3%)	
JL Elevations:				0.788
≤ 4 mm	466 (82.9%)	283 (85.2%)	183 (79.6%)	
> 4 mm	96 (17.1%)	49 (14.8%)	47 (20.4%)	
JL distalizations:				0.417
≤ 4 mm	266 (72.7%)	164 (74.2%)	102 (70.3%)	
> 4 mm	100 (27.3%)	57 (25.8%)	43 (29.7%)	

Values given as mean (standard deviation) or number (%). Measurements all given in millimeters (mm). Bold values indicate statistical significance

*JL* joint line

TKAs performed with a PS, MC, or CPS inserts were more likely to be elevated than TKAs performed with CR and UC inserts (Table 4). There were no significant differences in JL change or the breakdown of the directionality of change when grouping insert types depending on whether the PCL was resected or retained (Table 5). The four participating arthroplasty surgeons demonstrated differences in JL position change for both RA-TKAs and C-TKAs, when controlled for implant type between RA-TKAs and C-TKAs (Table 6).

**Table 4:** Comparison of joint line change based on polyethylene style

	CPS (*N* = 84; 58 RA-TKA, 26 C-TKA)		MC (*N* = 240; 169 RA-TKA, 71 C-TKA)		CR (*N* = 102; 61 RA-TKA, 41 C-TKA)		PS (*N* = 377; 222 RA-TKA, 155 C-TKA)		UC (*N* = 197; 90 RA-TKA, 107 C-TKA)		*p* value
JL change	− 0.35 (3.5)		− 0.40 (3.3)		0.23 (3.5)		− 0.73 (3.0)		0.76 (3.1)		** < 0.001**
	RA-TKA	C-TKA	RA-TKA	C-TKA	RA-TKA	C-TKA	RA-TKA	C-TKA	RA-TKA	C-TKA	
	− 0.27 (3.6)	− 0.55 (3.4)	− 0.05 (3.3)	− 1.3 (3.3)	0.31 (3.4)	0.11 (3.8)	− 0.48 (3.0)	− 1.1 (2.9)	0.94 (3.2)	0.61 (3.0)	
JL change directionality:											** < 0.001**
Elevation	51 (60.7%)		145 (60.4%)		49 (48.0%)		225 (59.7%)		93 (47.0%)		
	RA-TKA	C-TKA	RA-TKA	C-TKA	RA-TKA	C-TKA	RA-TKA	C-TKA	RA-TKA	C-TKA	
	34 (58.6%)	17 (65.4%)	100 (59.2%)	45 (63.4%)	31 (50.8%)	18 (43.9%)	123 (55.4%)	100 (65.4%)	44 (48.9%)	48 (44.4%)	
No change	5 (5.95%)		17 (7.08%)		5 (4.9%)		36 (9.5%)		9 (4.55%)		
	RA-TKA	C-TKA	RA-TKA	C-TKA	RA-TKA	C-TKA	RA-TKA	C-TKA	RA-TKA	C-TKA	
	3 (5.2%)	2 (7.7%)	11 (6.5%)	6 (8.5%)	2 (3.3%)	3 (7.3%)	29 (13.1%)	7 (4.6%)	2 (2.2%)	7 (6.5%)	
Distalization	28 (33.3%)		78 (32.5%)		48 (47.1%)		116 (30.8%)		96 (48.5%)		
	RA-TKA	C-TKA	RA-TKA	C-TKA	RA-TKA	C-TKA	RA-TKA	C-TKA	RA-TKA	C-TKA	
	21 (36.2%)	7 (26.9%)	58 (34.3%)	20 (28.2%)	28 (45.9%)	20 (48.8%)	70 (31.5%)	46 (30.1%)	44 (48.9%)	52 (48.1%)	

Values given as mean (standard deviation) or number (%). Bold values indicate statistical significance

*CPS* constrained posterior-stabilized, *MC* medial-congruent, *CR* posterior cruciate retaining, *PS* posterior stabilized, *UC* ultra-congruent

**Table 5:** Comparison of joint line change based on whether the PCL was retained or resected

	PCL retained* N = 232	PCL resected* N = 768	*p* value
JL position change	− 0.19 (3.5)	− 0.24 (3.2)	0.866
JL change directionality:			0.658
Elevation	129 (55.6%)	432 (56.3%)	
No change	14 (6.0%)	58 (7.6%)	
Distalization	89 (38.4%)	277 (36.1%)	

Values given as mean (standard deviation) or number (%)

*PCL* posterior cruciate ligament, *JL* joint line

*PCL retained in cases using a CR and some MC inserts (based on surgeon preference) and PCL resected in cases using UC, PS, CPS, and some MC inserts

**Table 6:** Comparison of arthroplasty surgeons and tibial inserts

	Surgeon 1		Surgeon 2		Surgeon 3		Surgeon 4		
	RA-TKAs (*N* = 150)	C-TKAs (*N* = 150)	RA-TKAs (*N* = 150)	C-TKAs (*N* = 90)	RA-TKAs (*N* = 150)	C-TKAs (*N* = 75)	RA-TKAs (*N* = 150)	C-TKAs (*N* = 85)	*p* value
JL change	0.68 (3.3)	0.52 (3.2)	− 1.4 (2.5)	− 2.00 (2.4)	0.50 (3.5)	0.28 (3.5)	0.05 (3.2)	− 1.37 (2.9)	** < 0.001**
Polyethylene styles:									
CPS	0 (0%)	1 (0.7%)	27 (18%)	9 (10%)	17 (11.3%)	11 (14.7%)	14 (9.3%)	5 (5.9%)	
MC	0 (0%)	0 (0%)	36 (24%)	26 (28.9%)	38 (25.3%)	30 (40%)	95 (63.3%)	15 (17.6%)	
CR	60 (40%)	41 (27.3%)	1 (0.7%)	1 (1.1%)	0 (0%)	0 (0%)	0 (0%)	0 (0%)	
PS	0 (0%)	0 (0%)	86 (57.3%)	54 (60%)	95 (63.3%)	34 (45.3%)	41 (27.3%)	65 (76.5%)	
UC	90 (60%)	108 (72.%)	0 (0%)	0 (0%)	0 (0%)	0 (0%)	0 (0%)	0 (0%)	

Values given as mean (standard deviation). Bold values indicate statistical significance

*JL* joint line, *CPS* constrained posterior-stabilized, *MC* medial-congruent, *CR* posterior cruciate retaining, *PS* posterior stabilized, *UC* ultra-congruent

## Discussion

This study found that on average RA-TKA better preserves the JL than C-TKA, although it is unclear that these differences are clinically meaningful. We found no significant differences between RA-TKAs and C-TKAs in the overall percentages of TKAs that had elevation, no change, or distalization of the JL. While there were a greater percentage of far-outliers in which the JL position was elevated more than 4 mm when using conventional methods, the differences did not reach statistical significance. Further, when looking at the changes in JL position, there were differences in JL position change depending on the tibial insert used and between the four surgeons for each technique.

To our knowledge, the current study is the largest to assess the JL change differences between primary RA-TKAs and C-TKAs. Additionally, the more restrictive inclusion criteria used eliminated some of the confounding variables that may have impacted results in prior studies. Particularly, other studies have included patients with flexion contractures as high as 30° [12, 13, 15, 17], whereas our study included patients with flexion contractures of no more than 10°. JL elevation is commonplace in knees with higher flexion contractures due to the need to proximalize the femoral component to enlarge the extension gap. Furthermore, while other studies included patients with as much as 20° of tibiofemoral coronal alignment [12, 13, 15, 17], ours included patients only if tibiofemoral varus was less than 10°. These pre-operative measurements have an influence on the results of JL position because of how they can affect operative decisions regarding orientation and depth of femoral bone resections.

The findings of the present study support the existing literature that robotic-assistance leads to better preservation of the JL than conventionally performed TKA [12, 13, 17, 19, 20]. We found that on average RA-TKA resulted in 0.04 (3.3) mm JL elevation, whereas C-TKA resulted in a mean JL elevation of 0.50 (3.2) mm. In our series, the JL was not elevated as much as other series in either C-TKA or RA-TKA, even when the PCL was resected, likely because of a concerted effort to control the JL position with both techniques. Also, with the particular implant used, the increased flexion gap resulting from PCL resection could be offset by a slightly larger femoral component, which would neutralize the flexion gap 2 mm. Therefore, offseting the increased gap observed with PCL resection and thus obviating the typical need to increase the extension gap to compensate. Vaidya et al. found an average of 0.9 (0.9) mm of elevation in RA-TKA and a 3.5 (1.6) mm of elevation for C-TKA when using PS TKAs (*p* < 0.001). Further, unlike our study which found 36.6% of total cases distalized, their’s found no cases of distalization [12]. Thiengwittayapron et al. reported mean JL elevations of 3.6 (3.3) mm for RA-TKA and 5.5 (0.4) mm for C-TKA using a single PS knee design (*p* = 0.004) [15]. When looking at the differences in JL position for the total sample of our study, 56.2% were JL elevations 2.4 (2.0) mm, 7.2% had no change, and 36.6% were JL distalizations 3.1 (2.1) mm. Similarly, Goh et al. found that after TKA there was radiographic evidence of JL elevation in 75% of cases, no change in 7%, and JL distalization in 18% [19].

When reviewing publications on JL changes, understanding how calculations are being performed and whether the differences include a factor of directionality or absolute values are being reported is useful because the direction of JL change may impact outcomes differently. Liow et al. reported a 1.9 (1.1) mm change in JL position for RA-TKA and a 3.5 (2.8) mm for C-TKA (*p* = 0.01) using a PS design, but these values are absolute values without specifiying directionality [17]. Likewise, Agrawal et al. found absolute JL changes of 0.334 (0.115) mm for RA-TKA and 2.304 (0.308) mm for C-TKA (*p* < 0.001) [13]. In that study, the authors used a combination of CR and PS knees, but did not sub-analyze their results based on implant type. Like our study, Popat et al. reported the mean changes in JL position of less than 1.0 mm for both RA-TKA and C-TKA (0.38 mm of distalization vs. 0.91 mm of elevation) [20]. It is unclear how the PCL was handled in that series. The larger variations in JL positional changes between studies likely relates to how pre-operative flexion contractures influence the JL positions after TKA, surgeon preferences on resection depths, with or without PCL resections, and the fact that some studies only reported JL elevations or average absolute differences without a function of directionality.

Prior studies comparing robotic-assistance and conventional techniques used a variety of methods of measuring JL position, relying on different anatomical landmarks when performing measurements [12, 13, 15, 17, 20]. These differences challenge the comparisons between studies and may explain variations in the absolute JL values. Multiple methods exist for quantifying the JL [20–23], but the present study utilized the AT method on both pre-operative and post-operative radiographs because of its strong reliability [18, 24, 25]. Further, the alignment and balancing techniques used for the RA-TKAs and C-TKAs must be accounted for because of their impact on JL position. Prior studies utilized various techniques between their RA-TKAs and C-TKAS such as mechanical axis alignment, measured resection technique, gap balancing technique, kinematic alignment, and functional alignment [12, 13, 15, 17, 20]. In our study, the RA-TKAs were performed with a restricted kinematic alignment algorithm in which the femoral component was positioned between 0° and 2° of varus relative to the mechanical axis, whereas the C-TKAs were performed with intra-articular guides, referencing a cut between 4° and 5° of anatomic valgus in all cases. It is unclear to what extent these differences in the orientation of the distal femoral resections had on joint line position between our two groups.

Implant design and PCL resection can influence JL position, and we analyzed whether the use of robotic assistance can mitigate their impact. Consistent with other studies [26], our findings show that on average TKAs performed with PS, CPS, and MC implants in which the PCL is resected are more likely to be elevated—in both RA-TKA and C-TKA—than those in which the PCL was preserved and CR implants utilized.

The effect of JL changes on functional and clinical outcomes remains a topic that warrants further investigation. While some studies argue that JL changes do not affect patient reported and clinical outcomes [27, 28], others have found significant relationships between JL changes and outcomes, particularly when the JL is elevated more than 4–5 mm [29]. In our study, 14.7% of RA-TKAs and 20.4% of C-TKAs had elevations more than 4 mm, and 25.8% of RA-TKAs and 29.7% of C-TKAs had distalizations more than 4 mm, so whether these outlier groups have compromised outcomes is the topic of ongoing investigation at our center.

Strengths of this study include a larger sample size and more stringent inclusion criteria, including minimizing coronal deformities and flexion contractures, all which could otherwise influence JL position. However, this was a retrospective, non-randomized study, which may be subject to inherent limitations such as variable techniques, patient selection, etc. Nonetheless, our groups were equally compared. Additionally, although it is a strength for this study that only a single robot and implant design were used, the results may not be generalizable to other robot systems or implant designs or individual surgeon techniques which may alter the outcomes of a similar analysis. Further, the subtle differences in establishing femoral component alignment and balance between the mechanical and restricted kinematic alignment techniques used in this study may have a subtle impact on JL positions. While a number of differences in JL position between groups in this study reached statistical significance, these differences were often less than 1 mm and are likely not clinically important. Future study should be performed to determine how JL position change affects functional outcomes and the need for manipulation under anaesthesia.

## Conclusion

RA-TKA better preserves the native joint line position than C-TKA. These findings are influenced by multiple factors, such as surgeon-specific techniques of femoral resections irrespective of use of robotics, the handling of the PCL and choice of polyethylene insert.

## Data Availability

The data will not be deposited in a public repository. Access to the data is limited by the privacy and data processing teams.
